# Development of a low-cost, remote plastic surgery skills training course during the COVID-19 Pandemic

**DOI:** 10.5116/ijme.63b4.081d

**Published:** 2023-01-20

**Authors:** Laura Awad, Benjamin Langridge, Edward Bollen, Zakee Abdi, Peter E.M Butler

**Affiliations:** 1 Charles Wolfson Center for Reconstructive Surgery, Royal Free Hospital, London, United Kingdom; 2 Department of Plastic Surgery, Royal Free Hospital, London, United Kingdom

**Keywords:** Surgical skill development, remote plastic surgery, surgery skills training, COVID-19 Pandemic

## Introduction

The global COVID-19 pandemic has presented many challenges across educational bodies, affecting how we deliver teaching and assess trainees. The pandemic has highlighted the need to maintain access to surgical skill development opportunities for surgical trainees. Increasing pressures, including reduced access to workplace opportunities, redeployment and limited availability of traditional training courses, presented significant obstacles to trainee progression. Simulation training is helping to bridge the gap left by the pandemic for surgical trainees, as well as enabling career progression and setting the foundations for skill development.

This article outlines the course design and highlights the obstacles encountered when delivering remote simulation. The efficacy of simulated bench-models for skills acquisition has been validated in multiple articles in the literature and provided the rationale behind the foundation structure of our course.^1^^-^^3^ This method has been shown to improve manual dexterity, multitasking, and overall surgical performance.

### Approach

The goal of this project is to promote high-quality and cost-effective surgical training which can be delivered globally during and beyond the COVID-19 climate. Our team has developed an "Essential and Advanced Plastic Surgery Skills Course". This can be delivered remotely, facilitated by online seminar platforms, using a low-cost "lab in a box" model and is intended to be delivered to trainees at the junior registrar or resident level. This course is now offered to both medical students and postgraduate surgical trainees at the University College of London.

One of the primary goals during the development of the course was to ensure this educational opportunity could be delivered remotely, enabling any trainee with an internet connection to access the skills training irrespective of global location. Remote learning reduces the financial and travel implications for surgical trainees.^4^ The development of a low-cost, reproducible, high-quality course has the potential to increase access to surgical training on an international scale.

### Our Solution

To deliver teaching in the learner's own environment, equipment was organised and distributed to each participant in the most compact and lightweight manner possible. Our "lab in a box" design provides at-home equipment for suturing, skin excision, local flap design, tendon repair and microsurgery. The equipment contained within the box included: a scalpel, suture pack, cork board, marker pen, bilaminar foam dressings, clear silicone sealant "tendon", sutures, microsurgical instruments, and a digital microscope. Costs were minimised by sourcing low-cost materials that could be distributed on a large, reproducible scale, available through online distributors.

Supplementary resources were made freely available, including videos of the key skills, and an instructional course handbook, all of which helped to reduce the financial burden on participants. Both the teaching and assessment were delivered through Zoom Video Communications using a two-device setup.

During the training course, continuous informal feedback was provided via video link. Formative assessment was composed of a global rating scale based upon the established Objective Structured Assessment of Technical Skills (OSATS), as well as task-specific criteria for each skill relating to the course curriculum (e.g., correct bite-size, space between sutures and knot tying).^5^^,^^6^ The global rating scale included participant positioning, instrument handling, tissue handling, and economy of movement. Each criterion was allocated a score from one to five, with one translating to poor performance and five translating to independent competency. After each exercise was completed, participants were requested to photograph their final model; this was subsequently reviewed by the instructors, with written feedback being provided to attendees following the conclusion of the course. Ensuring high-quality assessment and feedback was an important challenge to overcome. However, using the methods outlined above and ensuring adequate support for assessors, we were able to deliver a detailed assessment to surgical trainees.

### Evaluation

During its development, the course has continually been refined through iterative improvements and has received excellent feedback for the quality of equipment, audio-visual materials, quality of assessment, and feedback provided to trainees. Objective measures have demonstrated improvement in skill acquisition that is non-inferior to in-person training and assessment for junior surgical trainees. The course has received approval at the university level and is now incorporated into medical school curricula and specific MSc courses. Qualitative feedback received following a pilot course of 40 participants showed an overall positive response regarding confidence, quality of materials, teaching, and feedback received. 100% of participants would recommend this course.

Bilaminar foam dressings were used as a synthetic skin substitute; this material was found to be a low-cost alternative, and comparable to more expensive commercially available skin pads through both subjective questionnaire assessment by trainees and objective assessment of skin biomechanics through cutometry and durometry. Participants were able to learn suturing, skin lesion excision and local flap construction using this bilaminar material. Silicone sealant is a low-cost, inorganic alternative to biological materials and provides a suitable simulation of tendon repair. The use of clear sealant allows close inspection of the core suture and epitendinous repair, improving the quality of feedback provided by assessors and the overall participant experience.

For remote teaching via freely available video conferencing software, it was found that a dual-view electronic device set up to be optimal comprising of a laptop positioned opposite the student, and a mobile phone/tablet positioned on a stand above the workstation, orientated downwards. If required, the learner can engage sufficiently with a single device. This dual-device setup provides a view of the participants' worktops and the surgical positioning of the trainee. It allows the learner to engage with tutorship with the assessor and their peers. The supplementary course material was provided in the form of pre-recorded videos of skills, accompanied by an in-depth skills handbook which can be accessed freely on our departmental website.

Taking advantage of the learner's electronic devices, microsurgery skills can be taught remotely with the inclusion of a digital microscope which is a cost-effective, compact, lightweight alternative to the traditional binocular microscope. Traditionally, access to microsurgical skill development has been limited to specially designed workplace learning. Microsurgical courses often are extremely expensive, presenting a significant barrier to education, particularly in lower-resource environments.

Aspects such as depth perception were challenging to replicate in comparison to a table microscope; however, the digital microscope provided an excellent opportunity for surgical trainees new to microsurgery to develop their equipment handling, technique, and efficiency of movement. Positive feedback was received from 100% of the participants of our pilot course, who were all taught during the session and adequately performed microsurgical repair of a vessel and nerve using the device setup. Footage can be recorded by participants to enable post-workshop review and retrospective assessment and reflection. Alternatively, footage can be streamed for real-time interaction with a trainer.

The use of online platforms, such as Zoom Video Communications, Inc, facilitated the delivery of our course. This software is freely available for participants and provides added facilities such as screen sharing, breakout rooms and participant polls. Through our pilot, it was found that the optimal ratio of four students per assessor provided the highest quality yet scalable teaching and assessment. Participants were distributed through breakout rooms, and assessors rotated through the groups for each skill being trained. Platforms such as this provide built-in recording capabilities which were used for detailed assessments and further reviews with the participant after the course.

### Implications

This model of plastic and reconstructive surgical skills training overcomes many obstacles presented by the COVID-19 pandemic to surgical training, with high-fidelity materials in an easily distributable package. The development of low-cost, compact, lightweight materials was an important goal to increase accessibility for geographically remote trainees and to encourage continual skills development after course completion. Surgical simulation is a key method to employ for global surgical education during the COVID-19 pandemic and beyond. Our experience demonstrates that this can be delivered in a low-cost manner in contrast to the traditionally resource-intensive methods of simulation training.

### Conflict of Interest

The authors declare that they have no conflict of interest.
